# Behavioral classes related to physical activity and sedentary behavior on the evaluation of health and mental outcomes among Brazilian adolescents

**DOI:** 10.1371/journal.pone.0234374

**Published:** 2020-06-22

**Authors:** Fernanda Rocha de Faria, Valter Paulo Neves Miranda, Cheryl A. Howe, Jeffer Eidi Sasaki, Paulo Roberto dos Santos Amorim

**Affiliations:** 1 Department of Physical Education, Federal University of Viçosa, Viçosa, Minas Gerais, Brazil; 2 School of Applied Health Sciences and Wellness, Ohio University, Athens, Ohio, United States of America; 3 Department of Sports Science, Federal University of Triângulo Mineiro, Uberaba, Minas Gerais, Brazil; University of Kentucky, UNITED STATES

## Abstract

Latent Class Analysis can assist researchers interested in a better understanding of behavioral patterns and their association with health outcomes. This study aimed to identify lifestyle latent classes related to distinct domains of physical activity (PA) and sedentary behavior (SB) among adolescents and their association with health outcomes. This cross-sectional study included 217 Brazilian adolescents (15 to 18 years old, 49.3% female). The classes were based on moderate-to-vigorous physical activity (MVPA), light physical activity (LPA), number of steps, sedentary behavior (SB), and screen time (ST). To assess these behaviors, participants wore an accelerometer for one week. ST, demographic characteristics, socioeconomic status, and signs of common mental disorders (CMD) were evaluated through questionnaires. Latent Class Analysis was used to identify lifestyle classes. Three classes were recognized: “Active—Non-sedentary” (class 1) with 28.1% of adolescents; “Inactive—Non-sedentary” (class 2), 48.85%; and “Inactive—Sedentary” (class 3), 23.04%. Sex and signs of CMD were associated with the prevalence of the classes. Female adolescents presented 4.48 (95% CI 2.04–9.77) times more chance of belonging to the “Inactive—Sedentary” (class 3). Adolescents who presented CMD had 11.35 (95% CI 3.45–101.1) times more chance of belonging to the “Inactive—Non-sedentary” (class 2). The interaction between sex and signs of CMD showed that girls with signs of CMD were 9.20 (95% CI 1.17–71.52) more likely to belong to the Inactive—Sedentary class than the “Active—Non-sedentary”. Results indicate that sex and signs of CMD can affect the prevalence of the classes. Our findings highlight that physical inactivity and SB can be associated with signs of CMD, especially in female adolescents.

## Introduction

Physical inactivity and sedentary behavior (SB) are known as modifiable cardiovascular disease risk factors [1]. While physical inactivity is defined as not reaching the physical activity (PA) guidelines [2]; SB refers to activities with energy expenditure at the level of 1.0–1.5 metabolic equivalent units, performed in a sitting, reclining, or lying down positions, such as watching TV or using a computer [3]. These lifestyle behaviors are primary concerns among the pediatric population as these obesogenic habits developed during childhood usually track into adulthood [4].

Among Brazilian adolescents [5], more than half do not meet the current recommendation of 60 min of moderate-to-vigorous physical activity (MVPA) per day [6, 7]. The same unhealthy pattern applies to SB based on screen time (ST), as most Brazilian adolescents acquire considerable time on this activity [8] and exceed the recommendation of 120 min/day [9]. These lifestyle behaviors affect not only physical but also mental health, with studies reporting associations among physical inactivity, SB, and signs of common mental disorders (CMD), such as depression and anxiety [10, 11].

Most of the research involving lifestyle behaviors such as MVPA, SB and others (e.g. ST, light physical activity (LPA), and number of steps), have examined their association with outcomes in isolation. However, this type of research neither addresses the likelihood of the individual to be simultaneously involved in different lifestyle behaviors (e.g. watching TV while running on a treadmill), nor considers their interaction [12]. Throughout the day, these different lifestyle behaviors can co-occur [13] and commonly have a synergic harmful effect of increasing the prevalence of chronic diseases and mortality [14].

Recently, cluster analysis has been applied in different population targets to overcome this limitation and to explain the interplay among different lifestyle behaviors [13, 15–21]. Latent class analysis (LCA) is one type of clustering method and has emerged as an approach to assist researchers interested in a better understanding of behavioral patterns and their association with health outcomes [22]. The identification of classes with the same types of behaviors can help public policy to recognize those who present the highest health-related risk behavior and assist the development of interventions tailored to these specific groups.

There has been limited information about adolescents’ lifestyle-related to PA and SB through modeling techniques, mainly in Brazilians. Previous studies have involved a range of lifestyle behaviors in addition to MVPA and SB, such as sleep duration, physical violence, alcohol and tobacco use, and fruit and vegetable intake, among others [13, 15–21]. Moreover, out of these studies, just one study was carried out in Brazil but it was restricted to female adolescents [15]. Together, the findings of these studies imply that risky behaviors are prevalent and cluster together, with the majority of the adolescents being involved in unhealthy levels of MVPA and SB. However, we highlight that none of these studies have used accelerometer data to address lifestyle patterns related to different domains of PA and SB and their association with signs of CMD among adolescents. The purpose of this study was to identify adolescents’ lifestyle latent classes based on different domains of PA and SB, as well as their relationship with sex, signs of CMD, and socioeconomic and health variables.

## Materials and methods

### Study design and participants

This cross-sectional study was carried out between March and September 2018 from a random and representative sample of adolescents enrolled in the high school grades of the Federal Institute of Education, Science, and Technology of Triângulo Mineiro, Ituiutaba Campus, Minas Gerais, Brazil. The study protocol was conducted according to the guidelines in the Declaration of Helsinki and approved by the Research Ethics Committee involving human beings of the Federal University of Viçosa, under the decision number 74104217.3.0000.5153. Before conducting any measures, participants and their parents or legal guardians (when applicable due to participant’s age) provided written consent.

To calculate the sample size, we used a specific formula for cross-sectional studies contained in the EpiInfo software, version 7.2.2.16 (Georgia, United States). We set the population size at 471 (total number of students enrolled in the Institute high school grades) and the prevalence of outcome at 50% since the study considers multiple cardiovascular disease risk factors [23]. We adopted an acceptable error of 5%, a confidence level of 95%, and a design effect of 1.0. From these settings, we found a minimum sample size of 212 adolescents. We increased the sample size by 10% (21 adolescents) to recover possible losses, making up a total sample size of 233 adolescents. The sample was obtained through simple random sampling. Participants were representative of the grade and sex of the students attending the Institute.

To be included in the study, the adolescents were required to be between 15 and 18 years old, have returned the consent forms, and be regularly enrolled in a high school grade of the Institute. The exclusion criteria included pregnancy, temporary or permanent physical or mental disability, and regular use of diuretics/laxatives or the use of medication to control blood pressure.

The first author of this study performed all the measurements and was responsible for delivering and receiving the accelerometers. The data collection occurred over three meetings with each participant. At the first appointment, the selected adolescents were invited to participate in the survey, given information about the research and procedures, and received the consent and assent forms. The second meeting took place at the auditorium of the Institute, where the adolescents were expected to return the signed forms and received an accelerometer. A verbal explanation about the use of the monitor was given along with a leaflet with equivalent instructions. At the same meeting, they filled out the survey questionnaires, which took approximately 30 min to complete. Adolescents received a verbal description of the questionnaires before filling them out and were asked to answer honestly. In addition, we asked them to sit away from each other to maintain their privacy throughout the form filling. After 8 days, the third appointment took place in a private room designated by the Institute and lasted approximately 20 minutes. In this meeting, participants were expected to return the accelerometer and had their anthropometrics and blood pressure measured.

### Latent class manifest variables

We selected five latent variables to describe adolescents’ lifestyle classes related to different domains of PA and SB: MVPA, LPA, number of steps, SB, and ST. Variables were categorized dichotomously according to health recommendations (when available) to facilitate the interpretation of results.

### Physical activity, number of steps, and sedentary behavior—Accelerometer

MVPA, LPA, number of steps, and SB were measured by the ActiGraph accelerometer (GT3X model). The ActiLife software (version 6.13.4) (ActiGraph, LLC, Fort Walton Beach, USA) was used to perform all accelerometer analysis. Adolescents wore the monitors on their right hip on an elastic belt for 8 consecutive days, including during sleep at night. The adolescents were instructed not to change their daily routine and that the accelerometer was to be removed only for water-based activities, such as bathing and swimming. The first day of use (the day they received it) was not considered in the analysis to avoid the Hawthorne Effect [24].

We initialized the accelerometer to collect data at a 30 Hz sampling rate and used the normal filter. The data were reintegrated into 15-s epochs. Non-wear time was defined as consecutive zero counts/minute that lasted for at least 20-minutes. To be included in the analysis, participants were required to reach a minimum of 10 h.day^-1^ of “wear time” per day and at least 6 days a week, at least 1 of which was a weekend day. We evaluated daily graphs, inclinometer data, and converted these data into a Microsoft Excel comma-separated values (.csv) file to calculate the average sleep duration. These bed/wake times were used to create subject log diaries and removed from the analysis. Average sleep duration between 8 and 10 hours per day was classified as adequate sleep [25]. To classify PA and SB, we adopted the cut-points developed by Romazini et al. [26] validated for Brazilian adolescents using vector magnitude and 15-s epoch.

Based on the weekly average, adolescents were classified into specific behavior categories. MVPA was considered adequated when participants met 60 minutes per day [6, 7]. Due to the absence of a cutoff point for LPA and SB, the 75th percentile of the current dataset was used to classify these variables. The 75th percentile was also applied to the number of steps since just a small percentage of the participants (3.23%) met the cutoff point of 11,700 steps proposed by Tudor-Locke et al. [27].

### Screen time—Self-report

Participants were asked: *“On an average day*, *how many hours do you spend in front of any screen*?*”* Adolescents were told to consider all kinds of screens. ST was considered elevated when it was greater than 120 min, according to the guidelines for adolescents proposed by the American Academy of Pediatrics [9].

### Sociodemographic variables and health outcomes

Demographic characteristics included age, sex, and grade. The socioeconomic status was classified through a specific questionnaire suggested by the Brazilian Association of Survey Companies [28].

To assess CMD, we used the General Health Questionnaire, 12-item version [29], validated for application in Brazilian adolescents [30]. The instrument is easily applicable and suitable for assessing signs of depression and anxiety. It includes personal questions, such as: *“Have you been feeling unhappy and depressed*?*”*, *“Have you been able to enjoy your normal activities each day*?*”*, and *“Have you lost confidence in yourself*?*”* The responses of each question are coded by a four-point Likert scale to describe the presence and intensity of the CMD. The first two answers describe a normal mental state and were coded as "0". The last two responses indicate the presence of signs of CMD and were labeled "1". We totaled the scores from the 12 questions, and adolescents with a final score of ≥ 3 points were classified as *“with signs of CMD”* [31].

Alcohol and tobacco exposure was obtained by applying two modules from the short version of the Global School-Based Student Health Survey, validated for Brazilian adolescents [32]. The modules were evaluated separately and each one contains 6 questions. The adolescents answered questions like: *“How old were you when you first drank alcohol*?*”* and *“During the past 30 days*, *on how many days did you smoke cigarettes*?*”* For all questions, the first possible answer is the absence of consumption or exposure to alcohol or tobacco. The remaining response possibilities indicate some level of consumption or exposure. Final sums of the module equal to zero indicated were classified as *“non-exposed”*, while values ≥ 1 were interpreted as *“exposed”*.

Participants’ weight (kg) and height (cm) were measured by using a digital scale (Plenna, model Ice, São Paulo, Brazil) and portable stadiometer (Sanny Medical, São Paulo, Brazil), according to Lohman et al. [33]. Body mass index (BMI) was calculated through the formula (weight (kg)/height (m)^2^). BMI was classified in z-score, according to sex and age [34]. Waist and hip circumferences were measured using a flexible and inelastic measuring tape (Cardiomed, Curitiba, Paraná, Brazil). The waist circumference was measured horizontally at the umbilical scar and the hip at the buttock region, surrounding the largest circumference between the waist and the knees [35]. The waist-height ratio (WHtR) was calculated by dividing the average waist circumference (cm) by the height (cm). A value of WHtR ≥ 0.5 was considered an indicator of elevated cardiovascular risk.

Blood pressure was measured using an automatic device (Omron, model HEM 7113, Kyoto, Japan), according to the recommendations of the Guidelines of the Prevention of Atherosclerosis in Childhood and Adolescence [36]. Systolic and diastolic blood pressure were classified according to age, sex, and height percentiles [36].

### Statistical analysis

R Statistical Software [37] version 3.2.2 and IBM SPSS Statistics (IBM Corporation, Armonk, NY, USA) version 21 were used to perform the analyses. Alpha level was set at 0.05 to interpret the results.

LCA was used for modeling the “lifestyle” variable. This method is appropriate for analysis of interactions and associations between different kinds of behavioral variables. It is a person-centered approach and hence, can offer better conditions to evaluate heterogeneous and asymmetric variables, such as those related to the adolescents’ lifestyle [15]. LCA was conducted in the poLCA package (Polytomous Variable Latent Class Analysis) [38] available in the library of the R Statistical Software.

Diagnostic evaluation of the most parsimonious model was performed considering the Akaike Information Criterion (AIC), Bayesian Information Criterion (BIC), chi-squared goodness-of-fit test (χ2) and entropy. Model quality with the inclusion of covariates was evaluated by likelihood ratio tests (G^2^). The selection of the final model also considered the interpretability of the item-response probabilities of the manifest variables conditioned to the latent classes, based on the homogeneity and separation of the classes.

Kruskal Wallis test was used to verify the association of the following covariates in the prevalence values of the classes was verified: sex, signs of CMD, BMI, blood pressure, alcohol, and tobacco exposure. Bonferroni correction was used in the two-by-two post hoc tests to verify the difference between the k groups. Effect sizes were calculated to evaluate the differences between the continuous values of diastolic and systolic blood pressures among the three latent classes. The formula for the statistical tests of Mann-Whitney-U and Kruskal-Wallis-H were used to calculate η^2^. The effect sizes were classified according to the cutoff points suggested by Bakeman [39].

## Results

In total, 228 adolescents completed the survey, but 11 were removed from the sample for not using the accelerometer appropriately. Therefore, the sample comprised of 217 adolescents (16.08 ± 0.95 years old), of which 49.3% were female. Due to the small prevalence of underweight (1.38%) and obese (7.83%) BMI categories, they were grouped with eutrophic and overweight, respectively.

The majority of the adolescents (80.65%) wore the accelerometer for 7 days, while the remaining wore it for 6 days. Mean daily accelerometer usage time was 958 minutes (approximately 16 hours), without considering records during sleep time. Table 1 shows the absolute and relative frequencies of study variables.

**Table 1:** Sample characteristics, overall and by sex.

	Male (*n* = 110)	Female (*n* = 107)	Total (*n* = 217)
Age group			
**15–16**	73 (66.4%)	64 (59.8%)	137 (63.1%)
**17–18**	37 (33.6%)	43 (40.2%)	80 (36.9%)
Grade			
**10th**	45 (40.9%)	37 (34.6%)	82 (37.8%)
**11th**	36 (32.7%)	38 (35.5%)	74 (34.1%)
**12th**	29 (26.4%)	32 (29.9%)	61 (28.1%)
MVPA			
**Adequate (≥60 min/day)**	51 (46.4%)	25 (23.4%)	76 (35.0%)
**Insufficient (<60 min/day)**	59 (53.6%)	82 (76.6)	141 (65.0%)
LPA			
**Adequate (≥169.7 min/day)**	41 (37.3%)	13 (12.2%)	54 (24.9%)
**Insufficient (<169.7 min/day)**	69 (62.7%)	94 (87.9%)	163 (75.1%)
Number of steps			
**Adequate (≥8455 steps/day)**	40 (36.4%)	14 (13.1%)	54 (24.9%)
**Insufficient (<8455 steps/day)**	70 (63.6%)	93 (86.9%)	163 (75.1%)
SB			
**Adequate (<799.5 min/day)**	85 (77.3%)	78 (72.9%)	163 (75.12%)
**Elevated (≥799.5 min/day)**	25 (22.7%)	29 (27.1%)	54 (24.88%)
ST			
**Appropriate (≤2 hours/day)**	3 (2.7%)	2 (1.9%)	5 (2.3%)
**Elevated (>2 hours/day)**	107 (97.3%)	105 (98.1%)	212 (97.7%)
Sleep duration			
**Adequate (8–10 hours/day)**	12 (10.9%)	13 (12.2%)	25 (11.5%)
**Insufficient (<8 hours/day)**	98 (89.1%)	94 (87.9%)	192 (88.5%)
SES			
**Wealthy (classes A and B1)**	31 (28.2%)	22 (20.6%)	53 (24.4%)
**Middle (classes B2 and C1)**	68 (61.8%)	67 (62.6%)	135 (62.2%)
**Lower (classes C2 and D-E)**	11 (10.0%)	18 (16.8%)	29 (6.3%)
CMD			
**No signs of CMD (<3 points)**	47 (42.7%)	6 (5.6%)	53 (24.4%)
**With signs of CMD (≥ 3 points)**	63 (57.2%)	101 (94.4%)	164 (75.6%)
Alcohol			
**Non-Exposed (sum = 0)**	23 (20.9%)	12 (11.2%)	35 (16.1%)
**Exposed (sum > 0)**	87 (79.1%)	95 (88.8%)	182 (83.9%)
Tobacco			
**Non-exposed (sum = 0)**	69 (62.7%)	77 (72.0%)	146 (67.3%)
**Exposed (sum > 0)**	41 (37.3%)	30 (28.0%)	71 (32.7%)
BMI			
**Eutrophic (< 85th %)**	74 (67.3%)	83 (77.6%)	157 (72.4%)
**Overweight (≥ 85th %)**	36 (32.7%)	24 (22.4%)	60 (27.7%)
Blood Pressure			
**Adequate**	92 (83.6%)	107 (100.0%)	199 (91.7%)
**Elevated**	18 (16.4%)	0 (0%)	18 (8.3%)

MVPA, moderate-to-vigorous physical activity; LPA, light physical activity; SB, sedentary behavior; ST, screen time; SES, socioeconomic status; CMD, common mental disorders; BMI, body mass index.

Model fit statistics for two- to five-class solutions are provided in Table 2. The three-class model was chosen as the best fitting model for presenting consistent values of absolute and relative model fit, parsimony, homogeneity, and separation of classes. This model showed similar metrics with the two-class model, higher entropy value, and better indices compared to the other classes.

**Table 2:** Relative and absolute fit indices for latent class models.

Latent Classes	AIC	BIC	DF	χ^2^	G^2^	p-G^2^	Entropy
**2**	921.75	958.93	20	20.88	19.61	0.403	0.83
**3**	924.91	982.37	14	11.20	12.04	0.602	0.91
**4**	929.93	1007.67	8	3.92	5.06	0.750	0.74
**5**	938.02	1036.04	2	0.75	1.15	0.560	0.79
**6**	949.55	1067.84	-4	0.44	0.67	-	0.78

AIC, Akaike Information Criterion; BIC, Bayesian Information Criterion; DF, degrees of freedom; χ^2^, Pearson’s goodness-of-fit; G^2^, likelihood ratio deviance statistic.

**Fig 1:**
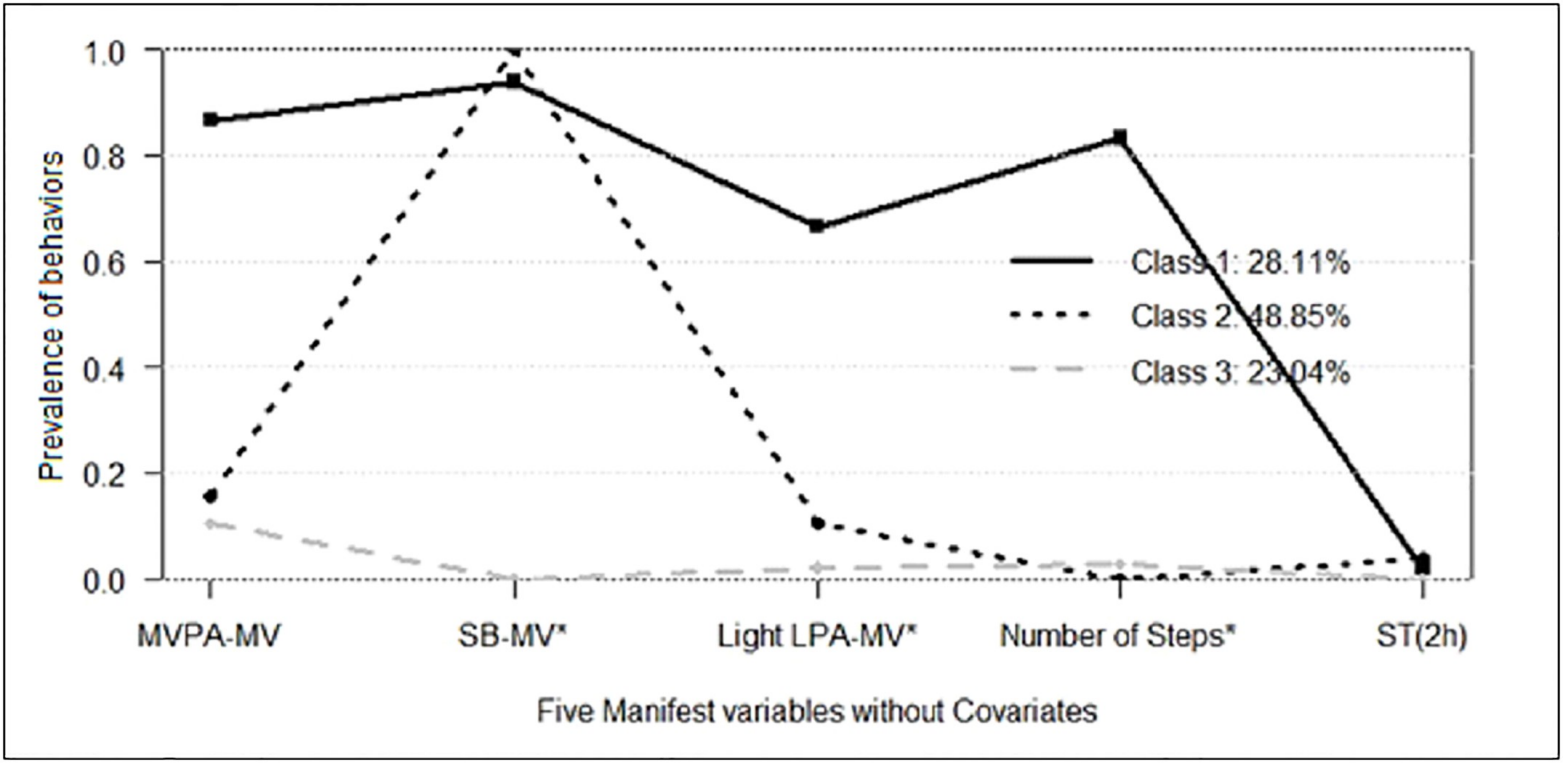
Distribution of participants across each of the three classes. MVPA, Moderate-to-vigorous physical activity; SB, Sedentary behavior; LPA, Light physical activity; ST, Screen time; MV, Magnitude Vector; *Variables measured with the accelerometer. Class 1: “Active—Non-Sedentary”, Class 2: “Inactive—Non-Sedentary”, and Class 3: “Inactive and Sedentary”.

After interpretation of the item response probabilities, the following three classes were identified: **Class 1**: “Active—Non-sedentary”; **Class 2**: “Inactive—Non-sedentary”; and **Class 3**: “Inactive—Sedentary”, as shown in Fig 1. The “Active—Non-sedentary” accounted for 28.1% (*n* = 61) of the sample. The majority of these participants were more likely to meet the MVPA recommendation (≥60 minutes) and to spend less time in SB (<75th percentile). The “Inactive—Non-sedentary” represented 48.85% (*n* = 106) of the sample. All the adolescents in Class 2 were characterized by less time spent in SB (<75th percentile) and low PA level. Class 3, labeled “Inactive—Sedentary”, comprised 23.0% (*n* = 50) of the sample. Adolescents in this class presented less than a 10% chance to reach the recommended MVPA (≥60 min), and a probability of approximately 0% of reaching adequate levels of the other health behaviors. Considering the total sample, they had the highest time spent in SB, and the lowest time spent in MVPA and LPA.

Table 3 presents continuous variables related to demographic characteristics, anthropometric measures, and cardiovascular risk factors among the three classes. Adolescents included in the “Inactive—Sedentary” class presented a higher CMD score than those assigned to the “Active and Non-sedentary” class. We highlight the diastolic blood pressure *p*-value (*p* = 0.06), which was close to the alpha level adopted in this study (*p*<0.05). After observing this, we performed a *Cohen’s d* effect size test on this variable and found a significant value of 0.263, which indicates the relative importance of the classes on diastolic blood pressure. Differences in systolic and diastolic blood pressures are displayed by the box plot graph in S1 Fig.

**Table 3:** Variation of age, cardiovascular disease risk factors, and CMD score among the latent classes.

Variables	Class 1 (*n* = 61) Active—Non-Sedentary		Class 2 (*n* = 106) Inactive—Non-Sedentary		Class 3 (*n* = 50) Inactive—Sedentary		p*
	Median	P25—P75	Median	P25—P75	Median	P25—P75	
**Age**	16.0	(15.0–17.0)	16.0	(15.0–17.0)	16.0	(15.0–17.0)	0.70
**BMI (kg/m**^**2**^**)**	21.4	(19.6–24.7)	21.1	(19.3–23.8)	20.4	(18.4–25.7)	0.32
**Hip (cm)**	97.1	(92.2–101.2)	96.8	(92.9–103.1)	95.9	(90.6–106.5)	0.74
**Waist (cm)**	76.6	(72.8–84.0)	75.4	(70.7–83.8)	73.8	(69.0–86.4)	0.20
**SBP (mmHg)**	105.5	(99.0–115.7)	102.5	(95.3–113.5)	104.2	(95.3–110.6)	0.27
**DBP (mmHg)**	56.0	(51.5–61.0)	57.2	(53.5–62.5)	59.5	(53.0–63.6)	0.06
**CMD score**	4.0^†^	(1.0–8.0)	6.0	(3.0–9.0)	6.0^†^	(3.7–10.0)	0.04*

*Significative p-value (<0.05) Kruskal Wallis test.

^†^Significative p-value (<0.016) of Bonferroni post hoc test between class 1 and class 3. BMI, body mass index; SBP, systolic blood pressure; DBP, diastolic blood pressure; CMD, common mental disorders.

We tested the effect of all possible combinations of covariates to the model above. After testing the covariates with the selected model, it was found that sex and signs of CMD were associated with the prevalence of the latent classes (Table 4). The new model with the inclusion of the covariates sex and signs of CMD showed that girls presented 4.5 (CI 95% 2.04–9.77) times greater likelihood of belonging to the “Inactive—Sedentary” (class 3) instead of the “Active—Non-sedentary” (class 1 –reference). In addition, adolescents who presented signs of CMD had 11.4 (CI 95% 3.45–101.1) times greater likelihood of belonging to the “Inactive and Non-sedentary” (class 2) than to the “Active and Non-sedentary” (class 1—reference) (Table 4). The association among classes and covariates is provided in Table 5.

**Table 4:** Sex and signs of CMD as predictors of membership in latent classes of adolescents.

α (Intercept)	Class 2 / Class 1					
	β (Coefficient)	SE	Odds Ratio	CI (95%)		p-value
Female†	0.02	0.63	1.02	0.36	3.49	0.97
Signs of Mental Disorders‡	2.43	1.12	11.35	3.45	101.10	0.05*
	Class 3 / Class 1					
	β (Coefficient)	SE	Odds Ratio	CI (95%)		p-value
Female†	1.50	0.40	4.48	2.04	9.77	0.003*
Signs of Mental Disorders‡	0.314	0.39	1.36	0.63	2.93	0.441

^†^Indicates that ‘male’ is the reference category.

^‡^ Indicates that ‘Without signs of CMD is the reference category.

*Significative association; SE: Standard error; CI 95%: Confidence Interval of 95%. Model fit values with covariates sex and signs of CMD: AIC: 902.01, BIC: 972.98, Pearson’s goodness-of-fit χ2 for multiway response profile table = 12.3, DF = 10, Likelihood ratio deviance statistic (G^2^) = 13.43 (p-value G^2^ = 0.200), and Entropy = 0.91.

**Table 5:** Association values of the latent class analysis model with individual covariates.

Covariates	Class 2 / Class 1‡						Class 3 / Class 1‡					
	β (Coefficient)	SE	Odds Ratio	CI (95%)		p-value	β (Coefficient)	SE	Odds Ratio	CI (95%)		p-value
Male†			1						1			
Female	1.09	0.71	2.97	0.74	11.94	0.150	1.97	0.46	7.17	2.91	17.63	0.001*
Without CMD†			1						1			
Presence of CMD	0.99	0.36	2.69	1.33	5.41	0.019*	2.37	1.09	2.97	1.27	90.01	0.05*
Eutrophic†			1						1			
Overweigth	-0.49	0.44	0.61	0.25	1.44	0.280	0.72	0.51	2.05	0.75	5.58	0.182
Adequate WHtR†			1						1			
Elevate WHtR	1.70	1.26	5.47	0.46	64.07	0.202	0.34	0.39	1.40	0.65	3.00	0.399
Normal BP†			1						1			
Elevated BP	-1.22	0.54	0.29	0.10	0.84	0.044*	-0.72	0.51	0.48	0.76	5.52	0.182
Alcohol—not exposed†			1						1			
Alcohol—exposed	0.30	0.50	1.34	0.50	3.59	0.550	-0.20	0.53	0.81	0.30	2.20	0.712
Tobacco—not exposed†			1						1			
Tobacco—exposed	0.28	0.64	2.24	0.37	6.61	0.664	-0.87	0.44	0.41	0.17	0.99	0.072
Adequate SD†			1						1			
Insuficcient SD	2.06	0.77	7.84	1.75	35.16	0.021*	0.52	1.25	1.68	0.14	19.49	0.682

^‡^ Reference class, Class 1;

^†^Reference categories;

*Significative association (p-value ≤0.05). Class 1: Active & Non-Sedentary; Class 2: Inactive & Non-Sedentary; Class 3: Inactive & Sedentary. CMD, common mental disorder; WHtR, waist-height ratio; BP, blood pressure; SD, sleep duration.

Lastly, we created a model to analyze the interaction between the covariates sex and signs of CMD. For this new model, the two-class model solution was chosen as the best fitting model, due to its consistent fit (AIC: 900.55, BIC: 947.46, χ^2^ = 21.33, Degrees of Freedom = 14, G^2^ = 21.27, p-value G^2^ = 0.214, and Entropy = 0.84). The two latent classes were labeled “Active and Non-sedentary” (γ = 28.11%) and “Inactive and Sedentary” (γ = 71.89%). The results showed that female adolescents with signs of CMD were 9.20 (95% CI 1.17–71.52) more likely to be in the “Inactive and Sedentary” class than the “Active and Non-Sedentary” class.

## Discussion

This study evaluated a cluster of five modifiable cardiovascular disease risk factors—MVPA, LPA, number of steps, SB, and ST—in a sample of Brazilian adolescents aged 15 to 18 years old. Our results contributed to a better understanding of adolescents’ behavioral patterns and their association with sex and psychological characteristics. Corroborating previous studies [13, 15, 16, 18], we found that healthy and unhealthy behaviors cluster together, and female adolescents presented a greater likelihood of being inactive and sedentary than males. A critical finding of this study was that physical inactivity and SB are associated with signs of mental disorders, especially in girls.

Regardless of the class, ST did not differentiate the groups because only a few adolescents complied with the 120-min/day limit recommendation [9] (Table 1). In spite of that, we maintained this variable in the model due to its harmful effects on physical and mental health, as has been previously highlighted by others [10]. This result suggested that interventions to fight the sedentary lifestyle pandemic must be primarily based on reducing ST in this population. By addressing this variable, interventions could reach the majority of the adolescents, as suggested by our results, which are in agreement with others that found a large amount of time spent by adolescents in this type of activity [8, 15, 18, 21]. The recommendation of restricting ST to less than 2h/day is well-known among researchers, but the high average time spent on this behavior (approximately 396 min per day) can suggest that the population who need this knowledge the most—adolescents—is not aware of this recommendation. Messages concerning ST should highlight the recommended time, its adverse effects on physical and mental health, and encourage the individual to exchange the excessive time spent on ST with healthier behaviors, such as PA of any intensity, or at least a standing posture. Since cell phone use or other small screens are a large portion of this excessive ST behavior, and since they are portable, incorporating even LPA during use can mitigate some of the negative consequences associated with this behavior.

Class 1, labeled “Active—Non-sedentary”, comprised approximately one-third of the sample and was considered the healthiest class in the study. Adolescents in this class were more likely to meet the MVPA recommendations (60 min per day) [6, 7], had the highest LPA and number of steps, and more than 90% of them reached the SB cutoff (<75th percentile). The CMD final score in this class was the lowest among the groups and was significantly lower than class 3 (“Inactive and Sedentary”) (Table 3). These results are in line with other studies which have shown a positive association between PA level and mental health [10, 11]. Furthermore, this class presented high levels of PA and SB based on ST, which confirms that these activities are not mutually exclusive, as pointed out by others [13, 15]. That is to say, high PA level does not replace SB and vice-versa. Interventions targeting this class must mainly focus on how to keep these individuals active and non-sedentary, and also on how to avoid the typical PA reduction and SB increase over the years, as reported by other authors [16].

The intermediate class -“Inactive and Non-sedentary”- was the largest group (n = 106) and had as its most significant feature the lowest time spent in SB compared to the other groups (<75th percentile). Despite that, adolescents in this class showed the smallest number of steps, and little time spent in MVPA and LPA. These results confirm the premise that one behavior does not replace the other; that is, low SB does not mean a high PA level. These findings imply that one healthy behavior does not imply in a healthy lifestyle. This physical inactivity pattern is the primary concern in this group due to its well-known harmful effects on general health [40]. Also, adolescents with signs of CMD had 11.4 (CI 95% 3.45–101.1) times greater likelihood of being in this class in relation to the “Active and Non-sedentary” class. These findings confirmed that signs of CMD have a meaningful effect on PA and SB patterns. We attributed these findings to the benefits of the PA level on mental health, as both of these classes presented practically the same SB pattern (Fig 1). Interventions tailored to this class must promote all domains of PA that can be accumulated throughout the day.

Class 3 (“Inactive and Sedentary”) was the worst behavior combination and was composed of 23.0% (n = 50) of the adolescents. This class exhibited the lowest levels of MVPA and LPA, and the highest levels of SB. This unhealthy pattern reflected on their mental health since adolescents in this group presented higher CMD final score than Class 1 (Table 3). The positive association among physical inactive, SB and psychological impairment in adolescents has previously been established [10, 11, 19]. Understanding the factors that make these individuals stand out from the others as the highest risk group may provide valuable direction for developing successful interventions.

In Brazil, there is also a high prevalence of anxiety and depression among adolescents. A national representative study of Brazilian adolescents, from 12 to 17 years old, applied the same CMD questionnaire and cutoff used in this study [41]. In that study, signs of CMD were prevalent in 33.6% of the participants from 15 to 17 years old, being higher among girls [41]. We highlight the higher prevalence found in our study, where approximately 75% of the adolescents were classified with signs of CMD (Table 1). We speculate that the reason for such difference may rely on the different school routine of our sample. Adolescents enrolled in this type of school take high school classes along with technical education. Their school routine includes 40 classes per week, from 7:30 am to 4:50 pm, with 17 required distinct subjects on average. Besides academic obligations, based on their grades, they can either apply to be a subject tutor or a research fellow, being involved in diverse types of activities during lunch break. These adolescents live as “mini-adults”, and such an exhausting routine full of pressure may be the justification for the high prevalence of signs of CMD found in our study.

Our results supported the well-established knowledge that females are more physically inactive and sedentary than males [17, 21]. Girls were 4.5 (CI 95%: 2.04–9.77) more likely to belong to the “Inactive—Sedentary” (class 3) instead of the “Active—Non-sedentary” (class 1). This fact shows female adolescents as an at-risk group and confirms our hypothesis of sex disparity related to PA and SB. However, our study went beyond that by evaluating two classes and extended the literature by showing the interaction between sex and signs of CMD. Females adolescents with signs of CMD were 9.2 (95% CI: 1.17–71.52) more likely to be in the “Inactive and Sedentary” class than in the “Active and Non-Sedentary” class. These results suggested that physical inactivity and SB can be associated with signs of CMD, especially in girls. These findings should not be disregard as adolescence is a period for the onset of behaviors that can be tracked into adulthood [17]. PA has been associated with improvements in mental health, mainly by increasing self-esteem [42] and reducing depression [10].

It is difficult to compare our results with others because just a few studies with adolescents between 15 and 18 years old have applied LCA, and most of them included different health behaviors on the model. One study worth mentioning was developed by Miranda et al. [15] and involved 405 Brazilian female adolescents from 14 to 19 years old. MVPA, number of steps, ST, sitting time, and number of meals were included in the LCA model. The variables clustered in three classes, two of which were also found in our study (“Inactive-Non-sedentary” and “Inactive and Sedentary”). The other class found by Miranda et al. [15] was the “Active and Sedentary”, despite the low frequency found in this group (6.2% of the sample). Unlike our study, which has found the “Inactive and Non-sedentary” as the largest class (48.9%), Miranda et al. [15] found that 77.5% of their sample were classified as “Inactive and Sedentary”. We speculate that the difference in our results should be explained by the sample involved. While we had both sexes, the other study restricted their sample to female adolescents, who are known to be more inactive and sedentary. Together, all these findings and prevalence mentioned above suggested that traditional Brazilian public policy to improve PA in adolescents, especially females, does not seem to be effective. More personal interventions with actions tailored to the specificity of each group may be a better option to promote healthy behaviors. Moreover, adolescents should learn and be educated about multiple health risk factors and their consequences in adulthood. Thus, the school environment should be seen as a critical place in combating youth risk behaviors. We state it because the majority of adolescents are enrolled in this context and spend most of their days in it. Active breaks, discussing groups, and a subject related to the adoption of healthy behaviors are some of the options to be developed in this context. The school can play a critical role not only in the adolescents’ intellectual development but also in the empowerment of their knowledge about health.

The strengths of this study include the uniqueness of the findings, the application of LCA to identify behavioral patterns, and the objective measurement of PA and SB. Besides that, the non-wear time of 20 minutes and the fact that most of the adolescents wore the accelerometer 7 days for 16 hours per day, on average, highlight the high adherence of the participants to the protocol and give reliability to the results. However, this study has some limitations. First, due to its cross-sectional design, we cannot make any conclusions about the causality of the associations. Second, the accelerometer was not used during water-based activities (e.g., swimming), which could lead to an underestimation of the PA level. Third, like all self-report methods, the answers to the questionnaires relied on the adolescent’s ability to accurately recall information. Lastly, a dichotomization of the variables to facilitate the interpretation may have led to the loss of some crucial information. In addition, it is worth mentioning that the findings would have been different if we had selected a distinct cutoff other than the 75th percentile for SB, LPA, and the number of steps. However, there is no consensus about a cut-point for the first two variables, and the majority of the adolescents did not reach the number of steps recommended by Tudor-Locke et al. [27]. Nevertheless, the 75th percentile was applied in another LCA study [15] and can be a useful method to compare adolescents with their peers. Future research should address the development of a SB cut-point and evaluate the distinct domains of PA and SB included in this study separately by sex. We also suggest longitudinal research to assess if the patterns are tracked into adult life and their effect on health over time. Finally, qualitative data should be sought to describe the determinants of each class (family, structure, mental, and others) that make adolescents adopt different behavior.

## Conclusions

In conclusion, three behavioral classes of different domains of PA and SB were found to represent the patterns of our sample. ST did not differentiate the classes and should be targeted as the main risky behavior in future interventions. Female adolescents had more chances to belong to the “Inactive and Sedentary”, while the ones with signs of CMD were more likely to be in the “Inactive and Non-sedentary” class. Therefore, holistic interventions must be developed, specific for each sex, and with particular attention to psychological health.

## Supporting information

S1 Fig (PDF)
