# Supplementary figures and images for: Behavioral classes related to physical activity and sedentary behavior on the evaluation of health and mental outcomes among Brazilian adolescents
Source: PLoS One. 2020 Jun 22;15(6):e0234374. doi: 10.1371/journal.pone.0234374 (PMC7307735; doi:10.1371/journal.pone.0234374)

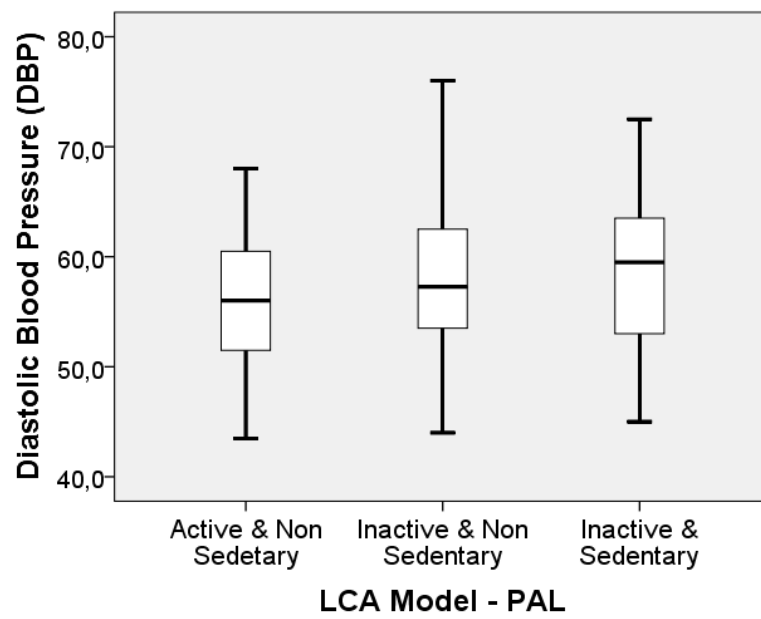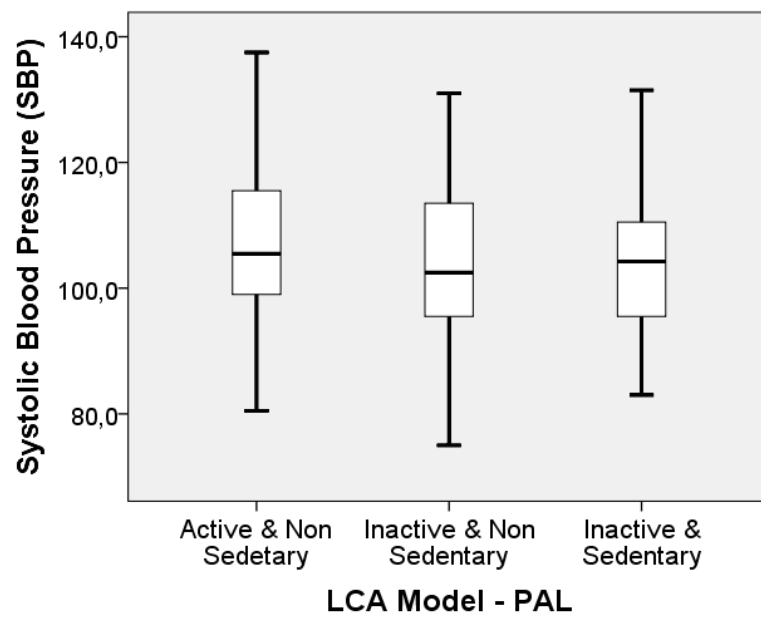

Supplement: S1 Fig — (PDF) [file pone.0234374.s001.pdf]
